# Supplementary material for: Optimization of injections with speculum-compatible devices to deliver ethyl cellulose-ethanol into the cervix to treat cervical dysplasia
Source: Sci Rep. 2025 Dec 20;16:2834. doi: 10.1038/s41598-025-32627-1 (PMC12824243; doi:10.1038/s41598-025-32627-1)
Supplement: Supplementary file 1 — Supplementary Material 1 [file 41598_2025_32627_MOESM1_ESM.docx]

**Title:** Optimization of injections with speculum-compatible devices to deliver ethyl cellulose-ethanol into the cervix to treat cervical dysplasia

**Authors:** Taya Lee*^1^, Vené Richardson-Powell*^1^, Gatha Adhikari^1^, Brian Crouch^2,3,4^, Nimmi Ramanujam^2,3,4,5^, Jenna Mueller^1,6,7^

*denotes authors contributed equally

^1^ Department of Bioengineering, University of Maryland, College Park, MD, USA.

^2^ Department of Biomedical Engineering, Duke University, Durham, North Carolina, USA.

^3^ Calla Health Foundation, Durham, North Carolina, USA.

^4^ Duke Global Health Institute, Duke University, Durham, North Carolina, USA.

^5^ Department of Pharmacology and Cancer Biology, Duke University, Durham, North Carolina, USA.

^6^ Department of Obstetrics, Gynecology & Reproductive Science, University of Maryland School of Medicine, Baltimore, MD, USA.

^7^ Marlene and Stewart Greenebaum Cancer Center, University of Maryland School of Medicine, Baltimore, MD, USA.

**Corresponding Author Information:** Dr. Jenna L. Mueller

**Email Address:** mueller7@umd.edu

**Telephone**: 301-405-8268

**Postal Address:**

3102 A. James Clark Hall

8278 Paint Branch Drive

College Park, MD, US, 20742

**Supplementary Figures**

**
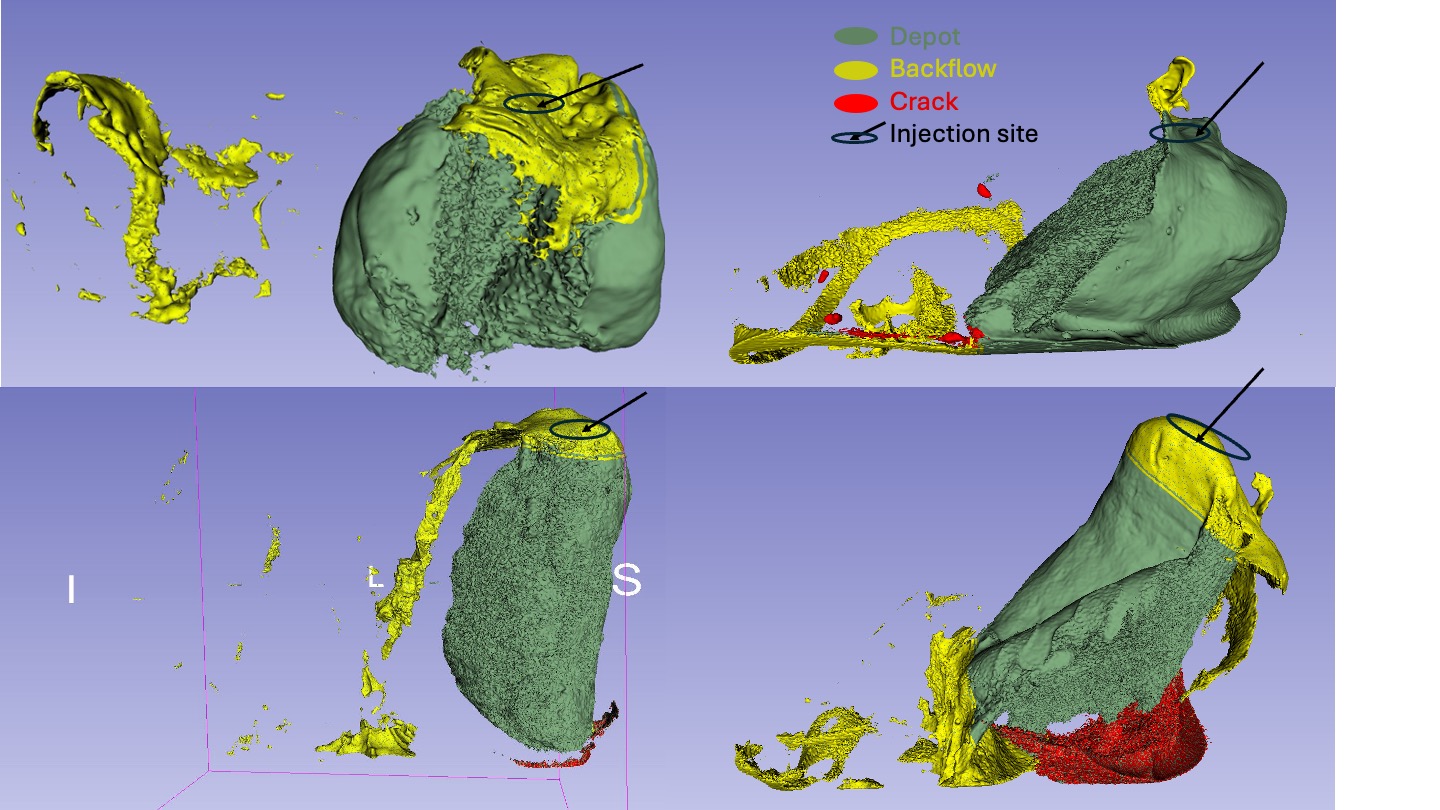
**

**Supplementary Figure 1.** Reconstructed excised swine cervical samples injected with 2mL of 6% ethyl cellulose-ethanol-iohexol. The single needle injector was used for samples on the left and needle extender device injector used for samples on the right. Yellow represents backflow and the black circle and arrow represent the injection site; the green represents the main depot region, red represents crack formation. The waterfall effect created from backflow can be visualized in yellow near at the injection site.
